# Long-Term Creep Behavior Prediction of Sol-Gel Derived SiO_2_- and TiO_2_-Wood Composites Using the Stepped Isostress Method

**DOI:** 10.3390/polym11071215

**Published:** 2019-07-20

**Authors:** Ke-Chang Hung, Tung-Lin Wu, Jyh-Horng Wu

**Affiliations:** 1 Department of Forestry, National Chung Hsing University, Taichung 402, Taiwan; 2 College of Technology and Master of Science in Computer Science, University of North America, Fairfax, VA 22033, USA

**Keywords:** activation volume, creep behavior, sol-gel process, stepped isostress method, wood-inorganic composites

## Abstract

In this study, methyltrimethoxysilane (MTMOS), methyltriethoxysilane (MTEOS), tetraethoxysilane (TEOS), and titanium(IV) isopropoxide (TTIP) were used as precursor sols to prepare wood-inorganic composites (WICs) by a sol-gel process, and subsequently, the long-term creep behavior of these composites was estimated by application of the stepped isostress method (SSM). The results revealed that the flexural modulus of wood and WICs were in the range of 9.8–10.5 GPa, and there were no significant differences among them. However, the flexural strength of the WICs (93–103 MPa) was stronger than that of wood (86 MPa). Additionally, based on the SSM processes, smooth master curves were obtained from different SSM testing parameters, and they fit well with the experimental data. These results demonstrated that the SSM was a useful approach to evaluate the long-term creep behavior of wood and WICs. According to the Eyring equation, the activation volume of the WICs prepared from MTMOS (0.825 nm^3^) and TEOS (0.657 nm^3^) was less than that of the untreated wood (0.832 nm^3^). Furthermore, the WICs exhibited better performance on the creep resistance than that of wood, except for the WIC_MTEOS_. The reduction of time-dependent modulus for the WIC prepared from MTMOS was 26% at 50 years, which is the least among all WICs tested. These findings clearly indicate that treatment with suitable metal alkoxides could improve the creep resistance of wood.

## 1. Introduction

Wood and wood-based composites have been widely used in everyday human lives. However, those materials have some disadvantage properties (e.g., dimensional instability, susceptibility to biological degradation, and flammability), thus limiting their exterior application and long-term utilization [1,2]. Over the past few decades, various wood modifications, including heat treatment, esterification, and inorganic modification by sol-gel technology, have been employed to improve their properties and to enhance their quality [3,4,5,6,7]. Among these, the sol-gel derived wood-inorganic composite (WIC) approaches have received a lot of attention over the last few years [8,9,10,11,12]. These WICs have been proven to be effective in improving the flame retardancy, thermal stability, UV stability, and fungal resistance compared to wood [10,13,14,15,16,17]. However, the creep property of WICs was rarely investigated.

Creep is one of the fundamental properties of materials limiting their long-term application as excessive deformation or reduced stiffness occurs over an extended period of time [18]. Therefore, the evaluation of creep behavior is essential in engineering applications. At realistic service-life durations, conventional creep tests are time-consuming [19]. To reduce the duration of a creep test, an accelerated creep test is required to obtain a master curve that is based on the superposition principle, consisting of the duration time, exposure temperature, and applied stress. Developed from the time‒temperature superposition principle (TTSP), the stepped isothermal method (SIM) can be used in the same manner as time-equivalence for a single sample to predict the long-term creep property of viscoelastic materials from stepped increments of temperature [20,21,22,23]. In the past few years, the stepped isostress method (SSM), which can evaluate the creep behavior of a single sample by a stepped increase of the stress level [24,25,26,27], was shown to be more beneficial in assessing the creep behavior of low-thermal-conductivity materials (e.g., wood and wood composites) compared to the SIM [28]. A previous study demonstrated that wood treated with methyltrimethoxysilane (MTMOS) could effectively enhance its creep resistance [29]. However, to the best of our knowledge, the effect of different metal alkoxides on the creep behavior of WICs has not been studied using the SSM. Therefore, in the present study, MTMOS, methyltriethoxysilane (MTEOS), tetraethoxysilane (TEOS), and titanium(IV) isopropoxide (TTIP) were used as precursor sols to prepare various sol-gel derived WICs, and the long-term creep behavior of all the WICs was predicted using the SSM.

## 2. Experimental

### 2.1. Materials

Japanese cedar (*Cryptomeria japonica* (L. f.) D. Don) sapwood (20–30 years old) supplied by the experimental forest of the National Taiwan University was used in this study. The MTMOS, MTEOS, TEOS, and TTIP were purchased from Acros Chemical (Geel, Belgium). All other chemicals and solvents used were of the highest quality available.

### 2.2. Preparation of Wood-Inorganic Composites

The oven-dried wood specimens, with dimensions of 3 mm (R) × 12 mm (T) × 58 mm (L), selected for this study were free of defects and exhibited a modulus of elasticity (MOE) of approximately 10.0 GPa to reduce material variability. Before the investigation, the samples were Soxhlet-extracted using a 1:2 (*v*/*v*) mixture of ethanol and toluene for 24 h and then washed with distilled water. The extracted wood samples were placed in an oven at 105 °C for 12 h, and their masses recorded. The WIC_TTIP_ was made directly from the oven-dried wood, while the wood used to make WIC_MTMOS_, WIC_MTEOS_, and WIC_TEOS_ was conditioned at 20 °C/65% RH for one week before preparation. The precursor sol was formulated with the desired metal alkoxide (i.e., MTMOS, MTEOS, TEOS, or TTIP), solvent (methanol or 2-propanol), and acetic acid at a molar ratio of 0.12/1/0.08 for preparing the WICs. The oven-dried or conditioned specimens were impregnated with the precursor sol for three days under reduced pressure. The impregnated specimens were then aged at 50 °C for 24 h and 105 °C for another 24 h [16,30]. The oven-dried weights of WICs were recorded to determine the weight percent gain (WPG).

### 2.3. Determination of Composite Properties

The density and flexural tests were carried out according to the ASTM [31,32] standards, respectively. The three-point static bending test with a loading rate of 1.28 mm/min and a span of 48 mm was used to determine the modulus of rupture (MOR) and the MOE of the specimens. Five specimens of each WIC were tested. All specimens were conditioned at 20 °C/65% RH for two weeks prior to testing.

### 2.4. Accelerated and Experimental Creep Tests

The universal testing machine (Shimadzu AG-10kNX, Tokyo, Japan) was used to implement the short-term SSM of wood and WICs for assessing the extended creep behavior. The creep strain at a reference stress is provided by Equation (1) based on the SSM:

ε(σ_r_, *t*) = ε(σ, *t*/α_σ_) (1)

where ε is the creep strain as a function of stress and time, σ_r_ is the reference stress, σ is the elevated stress, and α_σ_ is the shift factor. The SSM creep tests were conducted at isostresses between 30 and 80% of the average breaking load (ABL). Additionally, various SSM testing parameters were carried out to investigate the differences between the SSM creep tests. The stepped stress increments were 5%, 7.5%, 10%, and 12.5% ABL, and the dwell times were 2, 3, or 5 h.

All SSM tests were performed at 20 °C, which is below the glass transition temperature (*T*_g_), thus, the Eyring model (Equation (2)) is used to calculate the activation volume in this study [29]. This model was applied to estimate the shift factor (α_σ_), which shows the following express rate with the stress level [25,28]:

(2) $${\log\alpha }_{\sigma }=\log(\frac{\dot{\varepsilon }}{{\dot{\varepsilon }}_{r}})=\frac{V^{*}}{2.303kT}\left(\sigma -\sigma_{\mathrm{ref}}\right)$$

where $\dot{\varepsilon }$ and ${\dot{\varepsilon }}_{r}$ are respectively the creep rate at the elevated stress (σ) and reference stress (σ_ref_), V* is the activation volume, *k* is Boltzmann’s constant (1.38 × 10^−23^ J/K), and *T* is the absolute temperature.

On the other hand, a full-scale experimental creep test was performed as a basis for comparison to validate the master curves derived from the accelerated creep tests. Three specimens of each WIC were tested for creep at an applied stress of 30% ABL, and a linear variable differential transducer (LVDT) was used to measure and record the mid-span deflection values of the samples for a period of 120 days. All the samples during the experimental creep tests were held at 20 °C/65% RH.

### 2.5. Analysis of Variance

The software Statistical Package for the Social Science (SPSS 12.0) (Chicago, IL, USA) for the Windows program was used to perform statistical analysis. The significance of difference was calculated by Scheffe’s test, and *p* values < 0.05 were considered to be significant.

## 3. Results and Discussion

### 3.1. Flexural Properties of Wood-Inorganic Composites

The density and flexural properties of wood and WICs with WPG of 20% are shown in Table 1. The density of all five specimens ranged from 426 to 535 kg/m^3^. In addition, the MOE of all the WICs is in the range of 9.8–10.5 GPa, which is not significantly different from the untreated wood (10.0 GPa). According to Pasquini et al. [33], the stiffness of wood is found to depend mainly on the crystallinity of the cellulose. Therefore, the influence of sol-gel treatment on the crystallinity of the wood was the limit. However, the MOR of all the WICs increased to 93–103 MPa and is significantly higher than that of the wood (86 MPa) for all WICs except for the WIC_MTEOS_. The possible reason for the increase in MOR is the reaction of the inorganic compound with the wood via the sol-gel process to deposit or coat the cell wall, cell lumen, and intercellular space of the wood. However, further comparing the specific flexural properties in Table 1, the results showed that the sMOR, of all WICs, was not significantly different from that of untreated wood, while the sMOE of WIC_TEOS_ and WIC_TTIP_ was lower than that of untreated wood.

### 3.2. Accelerated Creep via SSM

**Figure 1:**
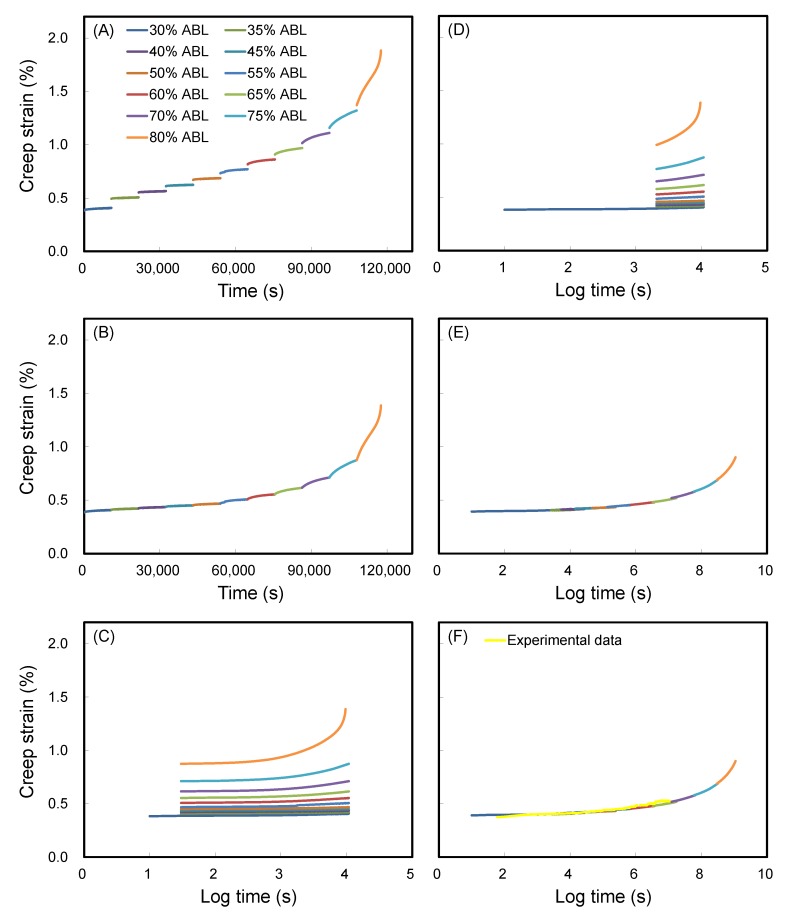
(**A**) The SSM creep data of the WIC_MTEOS_ (reference stress: 30% ABL; interval stress: 5% ABL; dwelling time: 3 h). The handing of the SSM test data for WIC_MTEOS_: (**B**) vertical shifting, (**C**) rescaled creep curves, (**D**) eliminating the period before the onset time of each stress step, and (**E**) horizontal shifting. (**F**) Experimental data and master curve.

**Figure 2:**
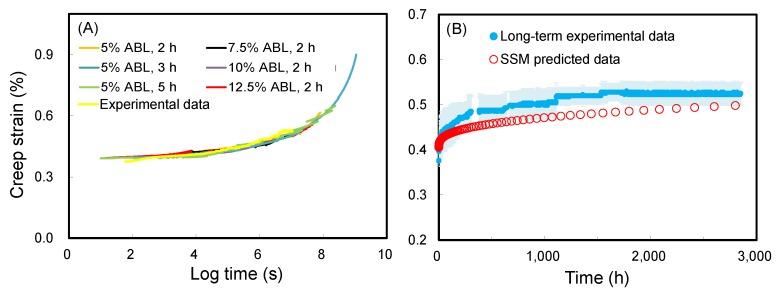
(**A**) Master curves of WIC_MTEOS_ from different SSM testing parameters at a logarithmic time scale. (**B**) SSM-predicted creep curve and experimental creep data of WIC_MTEOS_ at a normal time scale. Experimental creep data are displayed as the mean (blue line) ± SD (light blue ribbon) (*n* = 3).

**Figure 3:**
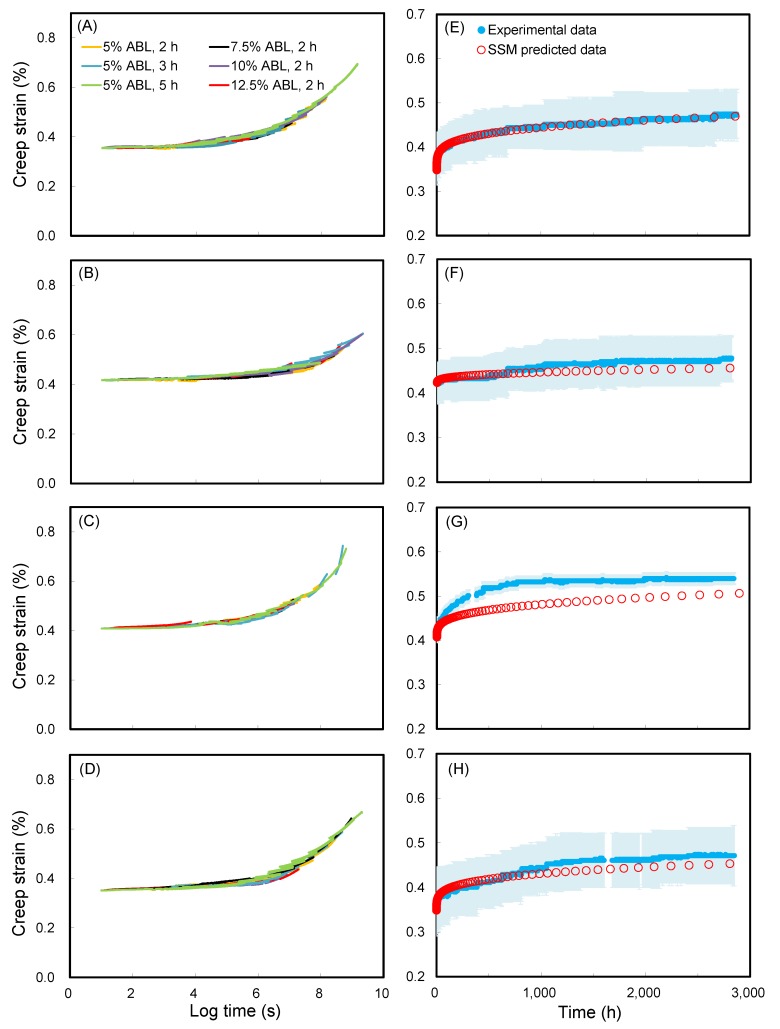
Master curves of (**A**) wood, (**B**) WIC_MTMOS_, (**C**) WIC_TEOS_, and (**D**) WIC_TTIP_, using different SSM testing parameters at a logarithmic time scale. The SSM-predicted creep curve and experimental creep data of (**E**) wood, (**F**) WIC_MTMOS_, (**G**) WIC_TEOS_, and (**H**) WIC_TTIP_ at a normal time scale. Experimental creep data are displayed as the mean (blue line) ± SD (light blue ribbon) (*n* = 3).

Generally, the SSM requires the following four adjustment steps to produce the master curve: vertical shifting, rescaling, eliminating, and horizontal shifting. In this section, for ease of understanding, the SSM creep curve of WIC_MTEOS_ at reference stress of 30% ABL with a 5% stepwise jump stress and a 3 h dwelling time was chosen as an example to outline the SSM and experimental creep tests. Figure 1A shows that an immediate strain jump between each load step was clearly observed in the SSM creep curve. However, there was no creep strain at each jump since the composites are elastic under instantaneous strain. Based on this, a vertical shifting is required to eliminate the elastic component in the recorded strain. This shifting links the end of the current loading curve to the start of the next loading curve at each load step, resulting in the continuous creep strain curve as shown in Figure 1B. The second step rescaling accounts for the deformation and damage from the stress and strain history of previous steps, and it was conducted by the modified method of Yeo and Hsuan [34]. As shown in Figure 1C, a series of independent creep curves were shifted along the logarithmic time axis to the reference stress level (30% ABL). Subsequently, the time before the onset time at the primary creep region was eliminated from each curve (Figure 1D), which is influenced by the history of the creep strain and the stress level. As a result of rescaling and eliminating, the master curve construction should be horizontally shifted along the time axis of the individual creep curves according to the shift factor log(*α*_σ_), where the magnitude of this shift factor is a function of the stress level. After this adjustment step, the final smooth master curve of WIC_MTEOS_ was produced (Figure 1E). Figure 1F shows that the SSM-fitted curve closely matches with the experimental data. The effects of the test parameters (stress increment and dwelling time) on the SSM master curves for WIC_MTEOS_ are presented in Figure 2. Clearly, the test conditions did not affect the master curve for a given WIC_MTEOS_ sample (Figure 2A), and these curves showed a high correlation to the long-term experimental creep behavior (Figure 2B). Similarly, the master curves of all the WICs had a similar trend with their long-term experimental creep data (Figure 3), and each individual master curve (SSM test result) could be used to predict its long-term creep behavior. Accordingly, SSM is a useful method for evaluating and comparing the creep behavior of newly developed materials.

**Figure 4:**
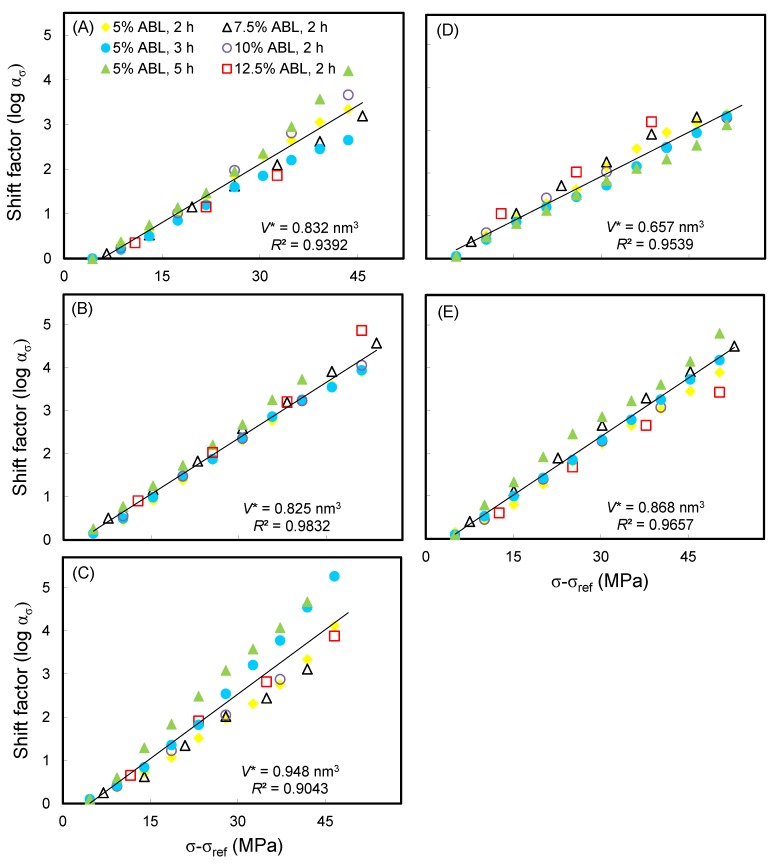
Typical Eyring plots of (**A**) wood, and WICs prepared from (**B**) MTMOS, (**C**) MTEOS, (**D**) TEOS, and (**E**) TTIP at a reference load of 30% ABL.

Additionally, a linear regression was used to determine the slope of the plot of the shift factor versus the stress level. Figure 4 shows the relationship between the shift factor and stress level, as validated by the values of the coefficient of determination (*R*^2^) being > 0.90. This result revealed that the superposition method used in the SSM approach was a valid approach to produce the creep master curve and that the same creep mechanism was active for each SSM test with different test parameters. The activation volume (V*) was calculated according to Eyring theory as Equation (2). The V* of all the WICs was in a range of 0.657–0.948 nm^3^. Of these, the WIC_MTMOS_ (0.825 nm^3^) and WIC_TEOS_ (0.657 nm^3^) were lower than that of the untreated wood (0.832 nm^3^).

### 3.3. SSM-Predicted Creep Curves

**Figure 5:**
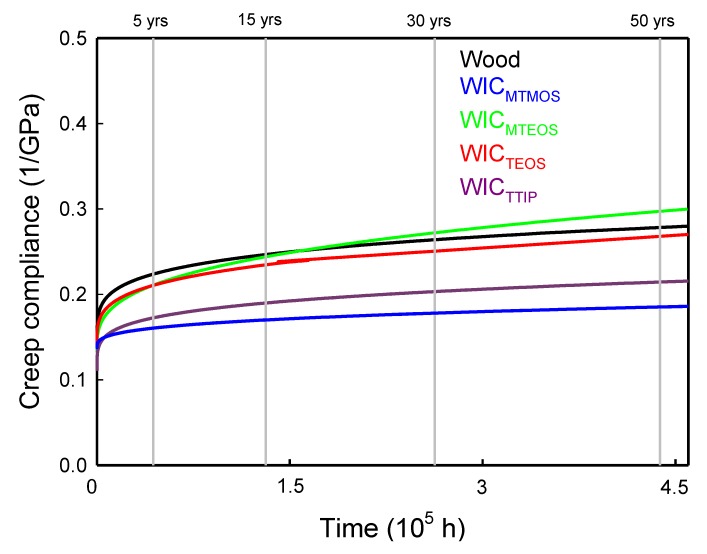
SSM-predicted creep data of wood and WICs prepared from MTMOS, MTEOS, TEOS, and TTIP.

The SSM-predicted compliance master curves of wood and WICs prepared from MTMOS, MTEOS, TEOS, and TTIP are presented in Figure 5. These results show that all the WICs had a lower creep compliance during the creep duration, except for WIC_MTEOS_. In addition, the creep master curves were fit to a Findley power law equation with three parameters, which is described by the following Equation (3):

*S*(*t*) = *S*_0_ + *at^b^* (3)

where *S*(*t*) is the time-dependent compliance value, *S*_0_ is the instantaneous elastic compliance value, *a* and *b* are constant values, and *t* is the elapsed time. The fitted parameters of the Findley power law model are shown in Table 2. It was seen that the model fits the SSM master curves of the WICs very well, all giving *R*^2^ values of greater than 0.99.

The instantaneous elastic compliances (*S*_0_) and the predicted time-dependent compliances (*S*(*t*)) of untreated wood and all WICs over the 5–50-year periods are listed in Table 2. The WIC_TTIP_ has the lowest *S*_0_ value (0.109 GPa^−1^), while the *S*_0_ value of untreated wood and all other WICs are in the range of 0.134–0.142 GPa^−1^. For the predicted compliance, the compliance values of the WICs were less than that of wood over a 50-year period, except for the WIC_MTEOS_. Among all WICs, WIC_MTMOS_ exhibited the lowest compliance values of 0.16, 0.17, 0.18, and 0.19 GPa^−1^ at 5, 15, 30, and 50 years, respectively. Miyafuji and Saka [35] pointed out that SiO_2_ with an even distribution in the cell walls was more effective in improving wood properties. Therefore, a possible reason for the above phenomenon is that the bulking effect of the MTMOS was higher than that of the other three precursor sols. In other words, during the sol-gel process, the MTMOS is mostly deposited in cell walls of wood rather than in cell lumens, which also results in a lower density of WIC_MTMOS_ under the same WPG as shown in Table 1.

Furthermore, the modulus reduction was calculated to evaluate the creep resistance of a sample under long-term conditions, and is given by Equation (4):

(4) $$\mathrm{Modulus}\;\mathrm{reduction}\;(\%)\;=\;\left[1-\frac{S_{0}}{S\left(t\right)}\right]\times 100\;$$

As listed in Table 2, the modulus of the untreated wood would decrease by 52% over 50 years. However, the modulus reduction of WICs decreased in a range of 26‒49% over a 50-year period, except for the WIC_MTEOS_ (55%). Of these, the least modulus reduction was found for WIC_MTMOS_ (26%). Accordingly, these results demonstrated that the creep resistance of the wood would be improved with MTMOS, TEOS, and TTIP treatment.

### 3.4. Accelerated Creep of Timber via SSM

**Figure 6:**
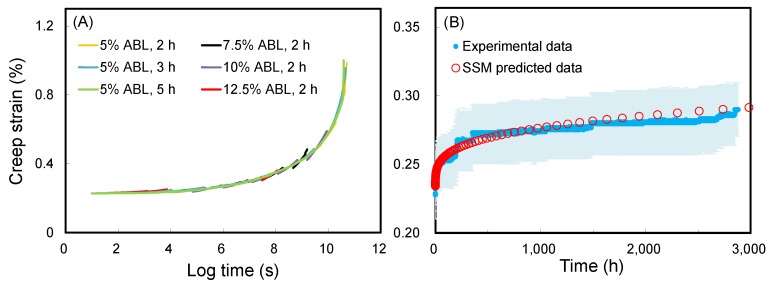
(**A**) Master curves of timber from different SSM testing parameters at a logarithmic time scale. (**B**) SSM-predicted creep curve and experimental creep data of timber at a normal time scale. Experimental creep data are displayed as the mean (blue line) ± SD (light blue ribbon) (*n* = 3).

To further understand and validate the suitability of the SSM approach for timber, the extended creep behavior was estimated from the Japanese cedar timber samples with dimensions of 12 mm (R) × 50 mm (T) × 230 mm (L) using the SSM method at a reference stress of 30% ABL. As shown in Figure 6, the test parameters did not affect the master curve for a given timber sample. The SSM-predicted creep curves match very well with the experimental creep behavior. The difference between the creep curve predicted by the SSM and the experimental creep of timber is very small and similar to the wood samples described above. Accordingly, the SSM can undoubtedly be applied for constructing the master curve of timber.

## 4. Conclusions

The extended creep behavior of various WICs, including WIC_MTMOS_, WIC_MTEOS_, WIC_TEOS_, and WIC_TTIP,_ were estimated using the stepped isostress method (SSM). The results of the SSM showed that it was suitably applied for constructing the master curves of wood and WICs. Accordingly, the WICs had lower creep compliance than the untreated wood during the creep duration, except for the WIC_MTEOS_. The reduction in time-dependent modulus of the untreated wood was 52% at 50 years, but after MTMOS, TEOS, and TTIP treatment, the reduction value decreased. Among WICs tested, WIC_MTMOS_ showed the least modulus reduction (26%) over a 50-year period. The activation volumes were 0.825, 0.948, 0.657, and 0.868 nm^3^ for WIC_MTMOS_, WIC_MTEOS_, WIC_TEOS_, and WIC_TTIP_, respectively. Overall, this study provided a reliable method for predicting the extended creep behavior of wood and various WICs.

## Figures and Tables

**Table 1:** Density and flexural properties of wood and wood-inorganic composites.

Specimen	WPG (%)	Density (kg/m^3^)	Flexural Properties		Specific Flexural Properties	
			MOE (GPa)	MOR (MPa)	sMOE (GPa)	sMOR (MPa)
Wood	‒	426 ± 35^b^	10.0 ± 1.1^a^	86 ± 3^b^	24.6 ± 2.6^a^	205 ± 7^ab^
WIC_MTMOS_	19.7 ± 0.7^a^	453 ± 18^b^	10.5 ± 1.7^a^	103 ± 12^a^	23.1 ± 2.5^ab^	226 ± 26^a^
WIC_MTEOS_	19.9 ± 1.1^a^	482 ± 29^ab^	10.1 ± 0.4^a^	93 ± 3^ab^	20.9 ± 0.8^ab^	193 ± 6^ab^
WIC_TEOS_	21.0 ± 1.5^a^	521 ± 44^a^	9.8 ± 0.4^a^	103 ± 6^a^	18.8 ± 0.7^b^	198 ± 12^ab^
WIC_TTIP_	20.0 ± 1.0^a^	535 ± 12^a^	10.1 ± 0.4^a^	101 ± 4^a^	18.9 ± 0.7^b^	188 ± 7^b^

Values are the means ± SD (*n* = 5). Different superscript letters (a and b) within a column indicate significant difference at *p* < 0.05.

**Table 2:** SSM-predicted creep compliances of wood and wood-inorganic composites.

Specimen	*S*_0_ (GPa^−1^)	*a*	*b*	*R* ^2^	*S*(*t*) (GPa^−1^)				Modulus Reduction (%)			
					Time (Years)				Time (Years)			
					5	15	30	50	5	15	30	50
Wood	0.134	0.0097	0.208	0.9982	0.22	0.25	0.26	0.28	40	45	49	52
WIC_MTMOS_	0.138	0.0008	0.317	0.9932	0.16	0.17	0.18	0.19	15	20	23	26
WIC_MTEOS_	0.134	0.0023	0.328	0.9911	0.21	0.24	0.27	0.30	36	45	51	55
WIC_TEOS_	0.142	0.0025	0.264	0.9925	0.21	0.24	0.25	0.27	34	41	45	49
WIC_TTIP_	0.109	0.0061	0.220	0.9942	0.17	0.19	0.20	0.21	37	43	46	49

*S*(*t*) = *S*_0_ + *at^b^*, where *S*(*t*) is the time-dependent compliance value, *S*_0_ is the instantaneous elastic compliance value, and *a* and *b* are constant values.
